# Carbohydrate Metabolism in the Intercaruncular Endometrium Is Affected by Form of Supplemental Selenium at Maternal Recognition of Pregnancy in Beef Heifers

**DOI:** 10.3390/ani15131903

**Published:** 2025-06-27

**Authors:** Sarah N. Carr, Benjamin R. Crites, Kwangwon Son, Phillip J. Bridges

**Affiliations:** 1 Department of Animal and Food Sciences, University of Kentucky, Lexington, KY 40546, USA; 2 School of Agriculture, Health, and Natural Resources, Kentucky State University, Frankfort, KY 40601, USA

**Keywords:** carbohydrate metabolism, glucose, intercaruncular endometrium, maternal recognition of pregnancy, selenium

## Abstract

Selenium (Se)-deficient soils result in Se-deficient forages that do not meet the nutritional requirement for this trace mineral in grazing cattle. Supplementation of Se conventionally occurs through a basal vitamin/mineral mix containing Se in an inorganic form (ISe), although organic Se (OSe) is available when cattle graze on forage. We previously reported that the provision of supplemental Se as a 1:1 mixture of ISe:OSe (MIX), in contrast to the industry standard of ISe alone, increases early luteal phase concentrations of systemic progesterone (P4) and increases the length of the conceptus at day 17 of pregnancy. The objectives herein were to determine the effect of the form of supplemental Se on (1) the abundance of mRNA transcripts encoding the family of selenoproteins in the ICAR endometrium; (2) systemic concentrations of glucose, triglycerides, and cholesterol; and (3) the global transcriptome of the ICAR endometrium. Commercial Angus-cross heifers (N = 20) were subjected to a 45-day period of Se depletion, followed by a 45-day period (Se repletion) where they were supplemented with ISe alone. They were then randomly assigned to receive a vitamin/mineral mix containing 35 ppm Se as either ISe (n = 10) or a 1:1 ratio of ISe and OSe (MIX, n = 10) for at least 90 days prior to synchronization of estrous. Following synchronization of estrous and artificial insemination, heifers were killed on day 17 of presumed pregnancy (maternal recognition of pregnancy, MRP) with their reproductive tracts and conceptuses recovered. When pregnancy was confirmed (n = 6 per treatment), ICAR biopsies were sampled from the uterine horn ipsilateral to the functional corpus luteum (CL) for targeted qPCR and RNA-Seq analyses. Targeted qPCR analysis revealed an effect of treatment (*p* < 0.05) on the abundance of one selenoprotein transcript (*Dio2*) during MRP. The quantification of serum parameters (d 0, 7, and 17) revealed treatment effects (*p* < 0.05) on systemic concentrations of glucose and cholesterol, but not triglycerides. RNASeq analysis revealed significant Se-induced changes in transcripts regulating carbohydrate metabolism. Overall, coinciding with the increase in conceptus length, the form of Se affected serum parameters during the establishment of pregnancy and intercaruncular transcriptomics during MRP.

## Simple Summary

Selenium (Se)-deficient soils result in forages that are deficient in this trace mineral. These forages do not meet the nutritional requirement for Se in grazing beef cattle. To overcome this, producers provide Se as a supplement, typically using an inorganic form (ISe), although organic forms of Se (OSe) are available when cattle graze on forage. This Se is incorporated into a class of proteins (selenoproteins) that function as potent antioxidants, protecting cellular membranes from the toxic effects of exposure to free radicals. We previously reported that heifers supplemented with a 1:1 ratio of organic and inorganic forms of Se (MIX), versus the industry standard of ISe alone, develop longer conceptuses during maternal recognition of pregnancy. This study was designed to investigate the relationship between the form of supplemental Se, the expression of selenoproteins in the intercaruncular (ICAR) endometrium, serum concentrations of glucose, triglycerides, and cholesterol, and the global ICAR transcriptome in heifers during this pivotal period in the establishment of a pregnancy.

## 1. Introduction

It has been well established that selenium (Se) should be provided as a supplement to grazing cattle in regions where the soils and forages are deficient in this trace mineral [1]. Producers conventionally supplement Se in a vitamin/mineral mix formulated with an inorganic form of Se (ISe, sodium selenite or sodium selenate), whereas when cattle naturally consume forage, organic forms (OSe, selenomethionine, and selenocysteine) are available [1]. Functionally, Se is an integral component of selenoproteins, a family of enzymes that include glutathione peroxidases [2], which exert protective antioxidant actions by catalyzing the conversion of cellular hydrogen peroxide (H_2_O_2_) into water (H_2_O [3]).

Studies in our laboratory have been designed to investigate the response to the form of supplemental Se provided as either ISe or a 1:1 mixture of ISe:OSe (MIX) to gain a Se-adequate status, on reproductive function. We initially reported a MIX (versus ISe)-induced increase in concentrations of systemic progesterone (P4) in the early luteal phase (days 6 and 7) of the estrous cycle [4,5]. This increased level of P4 appears to be the result of MIX-induced changes to the uptake of cholesterol by the low-density lipoprotein receptor in the corpus luteum (CL), and to a lesser extent, cholesterol generated from both de novo synthesis and the breakdown of cholesterol esters [4,6].

Increased early luteal phase P4 will affect endometrial development and its ability to support the growth of the post-hatch, pre-implantation conceptus [7,8,9]. Receptivity of the uterine endometrium to the conceptus is dependent on P4 [10], and P4-induced changes in endometrial transcriptomics have been shown to alter the composition of histotroph, which consists of growth factors, glucose, hormones, cytokines, enzymes, ions, adhesion molecules, and transport proteins that are secreted into the uterine lumen and are necessary for survival and growth of the conceptus prior to implantation [11,12].

Subsequently, we directly investigated the effect of the form of Se at the critical time of maternal recognition of pregnancy (MRP) and reported a MIX-induced increase in length of the conceptus at day 17 of pregnancy [13]. Concurrent to this, we observed a MIX-induced increase in the relative abundance of mRNA encoding the pivotal myostatin (MSTN) in the intercaruncular (ICAR) endometrium [13], a protein thought to increase glucose availability in histotroph [14]. Importantly, glucose sequestered from maternal blood and secreted into histotroph is the primary energy source for the post-hatch conceptus [11,12]. It appears that form of Se induced changes to the ICAR, with possible effects on serum metabolites, are affecting development of the early conceptus and its ability to signal MRP. The objectives herein were to determine, in MIX versus ISe supplemented heifers, (1) the relative differences in the abundance of mRNA transcripts encoding selenoproteins in the ICAR endometrium during MRP, (2) systemic concentrations of glucose, triglycerides and cholesterol at the time of insemination, as well as days 7 and 17 (MRP) of pregnancy, and (3) the global transcriptome of the ICAR endometrium at MRP. Understanding the mechanism(s) regulating the form of Se-induced changes in blood parameters and the ICAR endometrium at MRP is necessary to refine supplement recommendations to the producer.

## 2. Materials and Methods

All procedures were approved by the University of Kentucky’s Institutional Animal Care and Use Committee protocol number 2017-2828, date of approval on 14 December 2020.

### 2.1. Animals and Experimental Procedure

Angus-cross heifers (N = 20) underwent a 45-day period where they received a vitamin/mineral supplement with no added Se (depletion phase), followed by a 45-day period where they received 35 ppm Se as ISe to re-establish systemic Se to adequate concentrations (repletion phase) [15,16]. Heifers were then randomly assigned to one of two treatments: a vitamin/mineral mix containing 35 ppm Se as either inorganic Se (n = 10, ISe, sodium selenite, Prince Agri Products, Inc., Quincy, IL, USA) or a 1:1 ratio of ISe and OSe (n = 10, MIX, SEL-PLEX; Alltech, Inc., Nicholasville, KY, USA). Heifers were supplemented with their respective dietary treatment for at least 90 days prior to synchronization of estrous and insemination.

Whole blood was collected via jugular venipuncture throughout the duration of this trial (the depletion, repletion, and treatment phases). Whole blood was also collected at estrus, day 7, and day 17 of pregnancy. Concentrations of total blood Se were quantified in whole blood by the University of Kentucky’s Veterinary Diagnostics Laboratory (Lexington, KY, USA) using an Agilent 7900 inductively coupled plasma–mass spectrometer [17]. All animals maintained a Se-adequate status [15,16] throughout the treatment period of the trial; and there tended (*p* = 0.07) to be a greater concentration of Se in the blood of MIX compared to ISe supplemented heifers [13].

### 2.2. Experimental Regimen and Tissue Collection

Following at least 90 days of treatment with supplemental Se as either ISe or MIX, heifers were randomly injected with one or two doses of Lutalyse (25 mg dinoprost tromethamine, Zoetis, Parsippany, NJ, USA) to induce regression of the CL and then observed daily for behavioral estrus (d 0), using both visual means and with CowManager technology (Gerverscop 9, The Netherlands). The presence of a preovulatory follicle was confirmed via transrectal ultrasonography using a 5–8 MHz linear transducer (Ibex Pro, E.I. Medical Imaging, Loveland, CO, USA), with artificial insemination performed at 0, 12, and 24 h after the observation of estrus. All heifers were inseminated with commercially available frozen semen from a single bull with a record of high fertility.

The animals were slaughtered at the USDA-inspected University of Kentucky Meat Laboratory on d 17 of presumed pregnancy, and an intact conceptus was recovered from six heifers per treatment group (ISe, n = 6; MIX, n = 6). Only 6/10 heifers per treatment group were used for the analyses described herein. Endometrial biopsies were collected from the uterine horn ipsilateral to the ovary bearing the CL. This uterine horn was cut longitudinally to expose the lumen, and an 8 mm biopsy punch (Integra LifeSciences Production Corporation, Mansfield, MA, USA) was utilized to collect intercaruncular endometrial samples from each pregnant heifer. Endometrial samples were flash frozen in liquid N_2_ and then stored at −80 °C until RNA extraction, real-time PCR (qPCR), and RNA sequencing analysis.

### 2.3. Serum Analyses

On d 0, 7, and 17, approximately 8 mL of blood was collected into additive-free tubes (Vacutainer, Becton, Dickinson and Company, Franklin Lakes, NJ, USA) via jugular venipuncture for quantification of serum glucose, triglycerides, and cholesterol. These analyses were performed at the Cornell University Animal Health Diagnostics Center (Ithaca, NY, USA).

Serum concentrations of glucose were determined using the hexokinase method, which catalyzes the phosphorylation of glucose to form glucose-6-phosphate. This product is then oxidized by glucose-6-phosphate dehydrogenase to form 6-phosphogluconate, with the reaction also resulting in the conversion of NAD+ to NADPH, which is measured at λ = 340 nm; the product measured directly correlates to the concentration of glucose in the sample.

Serum triglycerides were quantified using the GPO-PAP method, an end-point reaction based on the disruption of triglycerides by lipoprotein lipase, resulting in NEFA and glycerol. Briefly, lipoprotein lipase catalyzes the hydrolysis of the triglycerides to yield glycerol and fatty acids. Following this, glycerol kinase catalyzes the phosphorylation of glycerol, and then glycerophosphate oxidase catalyzes the oxidation of glycerol-3-phosphate to hydrogen peroxide (H_2_O_2_) and dihydroxyacetone phosphate. The H_2_O_2_ product is measured at λ = 500 nm and is directly proportional to the concentration of triglycerides in the sample.

Total cholesterol was quantified using the CHOD-PAP method, an endpoint reaction. First, cholesterol and free fatty acids are released using cholesterol esterase, and then cholesterol is oxidized to cholest-4-en-3-one by cholesterol oxidase. During this second reaction, H_2_O_2_ is produced, which oxidizes a product that fluoresces at λex = 500/λem = 550 nm, a product that is proportional to the concentration of cholesterol present in the sample.

### 2.4. RNA Extraction

TRIzol (Invitrogen Corporation, Carlsbad, CA, USA) was used to extract total RNA from biopsy punches of the ICAR endometrium. The quality/quantity of all samples was determined using a NanoDrop ND-100 spectrophotometer (NanoDrop Technologies, Wilmington, DE, USA). All samples were revealed to have 260/280 absorbance ratios of 1.88 or greater.

### 2.5. RNA Sequencing

RNA-sequencing analysis was conducted on all 12 ICAR samples; library preparation was performed by Zymo Research Corporation (Irvine, CA, USA). Initially, total RNA (500 ng) was used to construct the total RNA-Seq libraries. Ribosomal RNA (rRNA) was removed as described in [18] with some modifications. Libraries were prepared using the Zymo-Seq RiboFree Total RNA Library Prep Kit (Zymo Research Corporation, Irvine, CA, USA). Sequencing of the RNA-Seq libraries was then performed using an Illumina NovaSeq with a sequencing depth of at least 30 million read pairs per sample.

The RNA-Seq pipeline, as used by the Zymo Research Corporation (Irvine, CA, USA), was adapted from the nf-core/rnaseq pipeline v1.4.2 [19] and built using Nextflow Di Tommaso, 2017; Nextflow enables reproducible computational workflows. FastQC v0.11.9 was used to determine the quality of raw reads, with adaptor and low-quality reads trimmed using Trim Galore! v0.6.6. Alignment of the resultant trimmed reads to the reference genome was performed using STAR v2.6.1d [20], with SAMtools v1.9 used for BAM file filtering and indexing [21]. Library quality control was executed using QualiMap v2.2.2-dev [22] and RSeQC v4.0.0 [23]. Duplicated reads were marked using Picard tools v2.23.9 (Broad Institute, Cambridge, MA, USA), and quality control of the duplication rate was analyzed using dupRadar v1.18.0 [24]. Preseq v2.0.3 [25] was used to estimate the complexity of the library; featureCounts v2.0.1 [26] was used to identify reads that overlapped with exons and to apply gene assignments.

**Figure 1:**
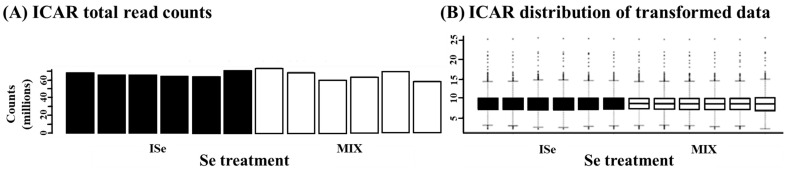
The (**A**) ICAR total read counts and (**B**) ICAR distribution of log2 transformed data derived from RNA-Seq analysis for each sample of ICAR tissue from heifers supplemented with a vitamin/mineral mix containing 35 ppm as ISe (n = 6) or a 1:1 mixture of ISe:OSe (MIX, n = 6).

**Figure 2:**
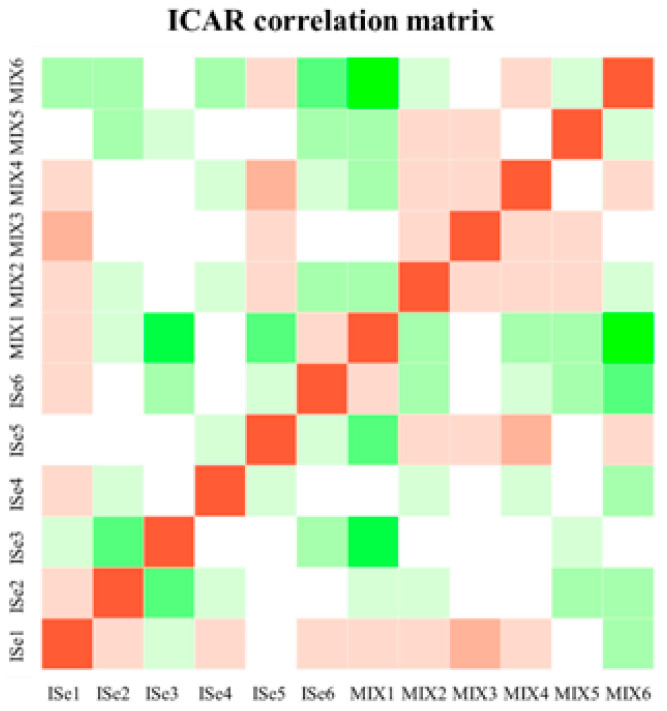
The ICAR correlation matrix of data derived from RNA-Seq analysis for each ICAR sample from heifers supplemented with a vitamin/mineral mix containing 35 ppm as ISe (n = 6) or a 1:1 mixture of ISe:Ose (MIX, n = 6). The average correlation among all samples was 0.96.

Count data was then uploaded to Integrated Differential Expression and Pathway Analysis (iDEP.96, [27]). Initially, count normalization analyses (count per million, CPM) were performed with lowly expressed transcripts removed at <1.0 CPM. Log10-transformed data were subjected to principal component analysis (PCA) and hierarchical clustering of all expressed genes to visualize sample variation. The average mapping percentage of all identified transcripts was 92.39%. Figure 1A reveals the total counts for all samples, and data for each normally distributed sample are shown in Figure 1B. The average correlation among all samples was 0.96 (Figure 2). Differentially expressed genes/transcripts (DEG) expression analysis was performed using DESeq2 v1.28.0 [28], which uses the Wald test for hypothesis testing by calculating the log2 fold change and dividing it by the standard error, resulting in a z-statistic that can be used to ascertain the *p*-value.

#### Functional Analysis

Global effects of the form of Se treatment on the abundance of transcripts in the ICAR during MRP were then assessed with DEGs identified in DESeq2 analysis and analyzed for canonical, functional, and network analyses using QIAGEN’s Ingenuity Pathway Analysis (IPA, QIAGEN, Redwood City, CA, USA). For each sample, the FASTQ file has been deposited into the National Center for Biotechnology Information Sequence Read Archive (accession number: PRJNA1096973).

### 2.6. Real-Time PCR Analysis

To quantify the relative abundance of mRNA encoding targeted genes from each ICAR sample, real-time PCR (qPCR) was used following a technique routinely reported from our laboratory [4,6,13,29]. Total RNA from each sample (~1 ug) was reverse transcribed into cDNA using SuperScript IV VILO Master Mix with ezDNAse Enzyme (Invitrogen by Thermo Fisher Scientific, Vilnius, Lithuania). A no-reverse transcription control for each sample was included to ensure qPCR results were not a result of contamination with genomic DNA.

The relative abundance of mRNAs encoding members of the family of selenoproteins was determined using a targeted qPCR analysis. We analyzed mRNA transcripts that encode the protein for three iodothyronine deiodinases (DIO1, DIO2, and DIO3), five glutathione peroxidases (GPX1, GPX2, GPX3, GPX4, and GPX6), three thioredoxin reductases (TXNRD1, TXNRD2, and TXNRD3), selenophosphate synthetase (SEPHS2), and other thirteen other identified selenoproteins (SELENOF, SELENOH, SELENOI, SELENOK, SELENOM, SELENON, SELENOO, SELENOP, SELENOR, SELENOS, SELENOT, SELENOV, SELENOW), as well as the selenoprotein P receptors (LRP2, LRP8 and TFRC).

The relative expression of mRNA transcripts identified from RNA-seq data associated with carbohydrate metabolism *(Adcy2, Aldob, Apoe, Bmp4, Edn1, Gaa, Gnaq, Manba, Mstn, Neu3, Pygl, Sgsh, Slc1a4, and Spp1*) and canonical Wnt signaling (*Cpt1a, Ctnnb1, Dkk1, Fzd6, Lrp5*, and *Lrp6*) was quantified by qPCR to corroborate results from the transcriptomic analysis. The NCBI Primer-BLAST tool (https://www.ncbi.nlm.nih.gov/tools/primer-blast/, accessed on 12 October 2023) was used to design all primers against their respective RefSeq. DNA sequencing of the target product cDNAs was verified by ACGT Inc. (Wheeling, IL, USA), and sequencing results were compared to each respective primer template using the NCBI Nucleotide-BLAST tool (https://blast.ncbi.nlm.nih.gov/Blast.cgi?PROGRAM=blastn&BLAST_SPEC=GeoBlast&PAGE_TYPE=BlastSearch, accessed on 12 October 2023). The GenBank accession numbers, forward and reverse primer sequences, amplicon length of each product and product identify for each transcript of interest are listed in Appendix A, Table A1, Table A2 and Table A3. For qPCR analysis, a total volume of 25 μL containing cDNA (5 uL), a 10 μM stock of each primer (1 uL forward and 1 uL reverse), 2 × SYBR Green PCR Master Mix (12.5 uL, iTaq Universal SYBR Green Supermix, BIO-RAD, Hercules, CA, USA), and nuclease-free water (5.5 uL) was used. Reactions were conducted using a Bio- Rad CFX Maestro thermal cycler (Bio-Rad, Hercules, CA, USA) using a 3-step amplification protocol with optimal annealing temperature determined for each primer pair.

The 2^−ΔΔCT^ method [30] was used to determine the relative abundance of each transcript using three constitutively expressed and normally distributed housekeeping genes that were not affected by Se-treatment: *Actb, Gapdh,* and *Hprt1*. Six heifers per treatment, in triplicate, were used for each transcript. Data were normalized to the relative expression level of the ISe-supplemented treatment group.

### 2.7. Statistical Analysis

The individual heifer was the experimental unit, and data are presented as least square means (±SEM). Data were analyzed for normal distribution and homogeneity. When appropriate, qPCR data were transformed for normalization, and each transformation is indicated in the results below the tables accompanying each data set. To determine the effect of the form of Se on the abundance of each mRNA transcript, data were analyzed using Student’s *t*-test (n = 6 per treatment) with the PROC TTest procedure of SAS statistical software package (version 9.4; SAS Institute, Inc., Cary, NC, USA). The relative expression for each transcript identified in RNA-sequencing results was subjected to the Wald test using DESeq2 as described above. For all data, results were considered statistically significant at *p* ≤ 0.05 or a tendency to differ at 0.05 < *p* ≤ 0.10.

## 3. Results

### 3.1. qPCR of Selenoproteins and Selenoprotein P Receptors in ICAR

Twenty-five selenoproteins and three selenoprotein P receptor transcripts were analyzed via targeted qPCR. We observed a decrease (*p* < 0.05) in the abundance of mRNA for only iodothyronine deiodinase 2 (*Dio2*) in ICAR from MIX- compared to ISe- supplemented heifers (Table 1). In addition, the relative expression of mRNA encoding the LDL receptor-related protein (*Lrp2*) tended (*p* < 0.1) to be decreased in MIX-Se supplemented heifers, and mRNA for iodothyronine deiodinase 1 (*Dio1*) was unable to be detected.

### 3.2. Serum Glucose, Triglycerides, and Cholesterol

**Figure 3:**
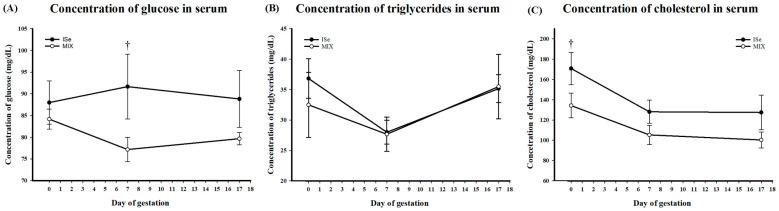
Concentration of (**A**) glucose, (**B**) triglycerides, and (**C**) cholesterol in serum from heifers at estrus (d 0) and gestational d 7 and 17 in heifers supplemented with either ISe (n = 6) or MIX (n = 6). (**A**) The dietary form of Se (*p* = 0.03), but not time (*p* > 0.05), affected serum concentrations of glucose. There was no interaction between the dietary form of Se and time (*p* > 0.05). (**B**) The dietary form of Se did not affect serum concentrations of triglycerides; however, an effect of time (*p* < 0.05) was observed. There was no interaction between treatment and time of systemic triglycerides (*p* > 0.05). (**C**) The dietary form of Se (*p* = 0.01) and day of gestation (*p* = 0.02) affected serum concentrations of cholesterol; there was no treatment x day interaction (*p* > 0.05) observed. Data were analyzed as an ANOVA with repeated measures. A † indicates a tendency to differ at 0.05 < *p* ≤ 0.01. Comparisons represented are between ISe and MIX at each indicated time point.

Concentrations of glucose, triglycerides, and cholesterol were quantified in the serum of all heifers on d 0 (estrus), and d 7 and 17 of pregnancy. Serum glucose was affected by treatment (Figure 3A). The dietary form of Se affected serum concentrations of glucose (*p* = 0.03), but time did not (*p* > 0.05), nor was there an interaction between the dietary form of Se and time (*p* > 0.05, Figure 3A). The dietary form of Se did not affect serum concentration of triglycerides (*p* > 0.05); however, there was an effect of time (*p* = 0.03), with no significant interaction between the two (*p* > 0.05, Figure 3B). Total circulating cholesterol was lower in the MIX vs. ISe treatment group with the main effects of treatment (*p* = 0.01), and an effect of day of gestation (*p* < 0.01), but no treatment x day interaction (*p* > 0.05, Figure 3C). In addition, a tendency for MIX to have a lower concentration of serum cholesterol on d 0 (170.83 ± 15.88 v 134.33 ± 12.22 mg/dL, *p* < 0.1) was observed, but no difference was observed on d 7 or d 17 of early pregnancy.

### 3.3. RNA-Sequencing in ICAR

#### 3.3.1. Cluster Analyses

**Figure 4:**
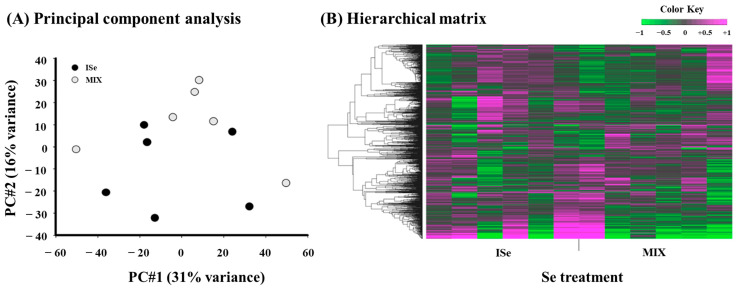
(**A**) Score plot and (**B**) hierarchical matrix of data derived from RNA-Seq analysis for each intercaruncular (ICAR) sample from heifers supplemented with either ISe (n = 6) or MIX (n = 6) in their vitamin/mineral mixes. (**A**) Principal components 1 and 2 (PC#1 and PC#2) accounted for 31% and 16% of the variance among samples, respectively. (**B**) Hierarchical cluster analysis comprised the top 1000 differentially expressed transcripts using iDEP.96.

To evaluate the relative relationships and variation among individual heifers, principal component analysis (PCA) of RNA-Seq data was performed. The score plot (Figure 4A) reveals that principal component 1 (PC#1, *x*-axis) explained 31% of variance among the samples and principal component 2 (PC#2, *y*-axis) explained 16% of variance. Results indicate that the ISe-supplemented heifers are less closely clustered than the MIX. Hierarchical clustering analysis of the DEGs (Figure 4B) revealed a considerable separation between treatments (ISe versus MIX), but there may be some overlap in transcriptomic profiles of the ICAR, consistent with PCA results.

#### 3.3.2. Differentially Expressed Genes

The Wald test of DESeq2 was used to determine changes in the abundance of ICAR transcripts between the ISe- and MIX-supplemented heifers. A total of 838 DEGs, with a total of 427 transcripts upregulated and 411 transcripts downregulated, were identified in MIX vs. ISe-supplemented heifers at *p* < 0.05. The differentiated genes that are most up-regulated and down-regulated in MIX compared to ISe are provided in Table 2.

#### 3.3.3. Pathway and Gene Network Analysis

**Figure 5:**
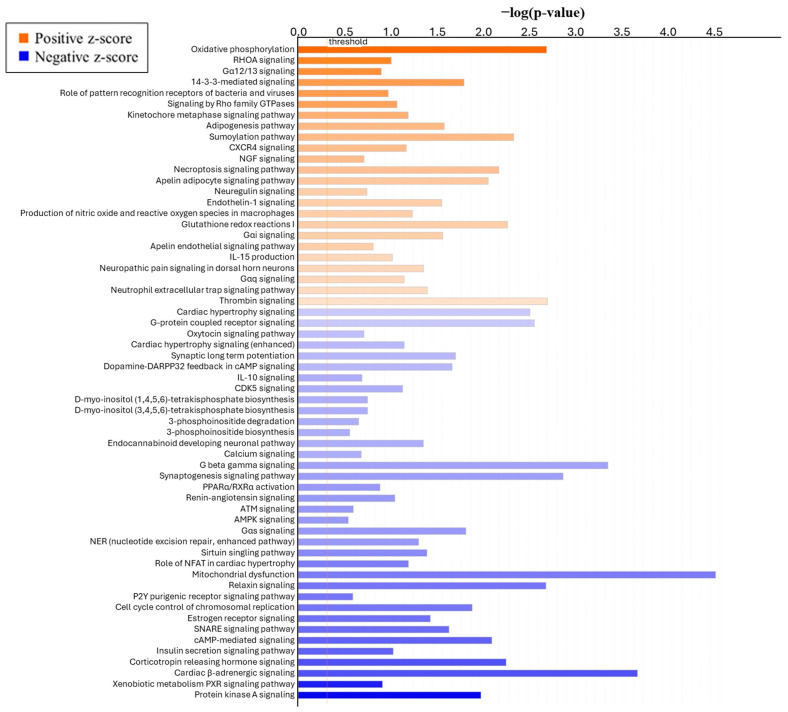
Top canonical pathways of genes differentially expressed from ICAR of heifers supplemented with either ISe (n = 6) or MIX (n = 6) in their vitamin/mineral mixes, as identified by IPA. Pathways are ranked by z-score, with orange (positive z-score) indicating a pathway that is predicted to be upregulated and blue (negative z-score) indicating a pathway that is predicted to be downregulated.

To determine the effect of the form of Se on global changes in DEGs, bioinformatic analysis was performed using QIAGEN’s Ingenuity Pathway Analysis (IPA, QIAGEN, Redwood City, CA, USA). The canonical pathway analysis revealed the top five pathways (Table 3) based on *p*-value that were affected by the form of Se are mitochondrial dysfunction (*p* < 0.0001), cardiac-adrenergic signaling (*p* < 0.0001), endocannabinoid neuronal synapse pathway (*p* < 0.0001), G beta gamma signaling (*p* < 0.0001), and synaptogenesis signaling pathway (*p* < 0.0001). Further, Figure 5 shows the top canonical pathways in ICAR ranked by z-score. Interestingly, the most positively affected pathways are oxidative phosphorylation (z-score = 3.00), ras homolog family member A (RHOA) signaling (z-score = 2.449), Gα12/13 signaling (z-score = 2.236), and 14-3-3-mediated signaling (z-score = 2.000). The most negatively affected pathways based on z-score are protein kinase A signaling (z-score = −2.324), xenobiotic metabolism PXR signaling pathway (z-score = −2.121), cardiac β-adrenergic signaling (z-score = −1.667), and corticotropin-releasing hormone signaling (z-score = −1.667).

The top upstream regulators identified using IPA were beta-estradiol, tumor protein p53 (TP53), GLI family zinc finger 1 (GLI1), histidine-rich glycoprotein (HRG), and Wnt family member 3a (WNT3a). The top 5 molecular and cellular functions with specific actions identified by IPA are indicated in Table 4. Only functions with a z-score of greater than or equal to the absolute value of 0.5 are reported. Additionally, key transcripts associated with carbohydrate metabolism and canonical Wnt signaling are reported in Table 5.

#### 3.3.4. RNA Sequencing Corroboration Using qPCR Analysis

To corroborate the findings of the RNA-seq analysis, qPCR was conducted on select transcripts associated with carbohydrate metabolism and canonical Wnt signaling during MRP (Table 5). A full list of gene transcripts associated with carbohydrate metabolism that were affected is listed in Appendix B, Table A4.

## 4. Discussion

We previously reported that the length of the preimplantation conceptus during MRP was increased in heifers supplemented with a 1:1 mixture of ISe:OSe (MIX) versus those that received the industry standard of ISe alone [13]. Concurrent to this, we reported a significant increase in the relative abundance of mRNA for *Mstn* [13]. MSTN protein is thought to increase the availability of glucose [14], the primary energy source for the post-hatch preimplantation conceptus [11], which plausibly provides a relationship between this transcript in ICAR and the observed MIX-induced advancement in conceptus development.

To expand upon these findings, we aimed to define the effects of form of supplemental Se (treatment) on (1) the abundance of mRNA transcripts encoding the functional selenoproteins in the ICAR endometrium during MRP, (2) circulating concentrations of glucose, triglycerides and cholesterol at the establishment of pregnancy, and (3) the global transcriptome of the ICAR endometrium.

### 4.1. Selenoproteins and Selenoprotein P Receptors in ICAR

We determined the effect of the form of Se required to yield a Se-adequate status on the abundance of mRNAs encoding the 25 identified mammalian selenoproteins and 3 selenoprotein P receptors [3,31] in the ICAR of heifers at d 17 of gestation (MRP). We hypothesized that multiple selenoprotein transcripts with known antioxidant capabilities, such as the glutathione peroxidases, would be significantly more abundant in MIX- versus ISe-supplemented heifers. We only observed a significant effect on the expression of mRNA encoding the intracellular iodothyronine deiodinase 2 (*Dio2*), which was decreased (*p* < 0.05) in MIX- versus ISe-treated heifers. This was an unexpected finding, especially given that this same experimental paradigm resulted in the differential expression of multiple selenoprotein transcripts in the CL [4]. It is, however, consistent with the form of Se effects on the expression of selenoprotein mRNAs in the caruncular endometrium, in which MIX-induced decreases in the abundance of only *Dio2* and *Dio3* were observed [29]. Iodothyronine deiodinases (DIOs) regulate the activity of thyroid hormones by activating and deactivating specific circulating and intracellular thyroid hormones. The regulatory actions of DIOs affect constitutive processes including thermogenesis, cell differentiation and proliferation, energy metabolism, and growth, thus affecting the regulation of metabolism of carbohydrates, proteins, and lipids [32,33,34]. It appears that a subtle form of Se effects on thyroid metabolism in the preimplantation uterine environment exists, noting that a high level of placental DIO3 expression is believed to protect the growing fetus from the activity of maternal thyroid hormones [35]. Of note, low levels of serum T4 can result in an increase in DIO2 activity [36], which may suggest lower systemic concentration of T4 in ISe compared to MIX-treated heifers. Unfortunately, we were unable to quantify circulating thyroid hormone concentrations due to limited sample availability in the present study.

### 4.2. Serum Glucose, Triglycerides, and Cholesterol

We investigated the effects of treatment (dietary form of Se) on systemic concentrations of glucose, triglycerides, and total cholesterol on d 0 (estrus) and on d 7 and d 17 of gestation. We hypothesized that the concentration of systemic glucose would differ with the form of Se treatment. As the primary source of energy for the post-hatched conceptus prior to implantation and formation of the placenta, glucose is sequestered from the maternal blood and secreted into histotroph [11]. Therefore, changes in glucose availability can have a tremendous impact on conceptus elongation and implantation. Serum concentrations of glucose were higher in ISe compared to MIX-treated heifers. Notably, Moraes et al. [37] observed significantly less plasma glucose on d 17 of gestation in high fertility compared to infertile heifers, with heifers classified as sub-fertile having an intermediate concentration of plasma glucose. The previously observed increase in mRNA abundance for *Mstn* in MIX-Se form heifers could be a tissue-specific metabolic change to account for the lower amount of this substrate in the blood. Myostatin has been shown to regulate glucose metabolism by promoting glucose uptake, supporting glycolysis, and decreasing the storage of glucose as glycogen [38], with increased MSTN also associated with an increase in glucose in histotroph [14].

We did not observe the expected form of Se-induced difference in the circulating concentration of triglycerides. With a backbone of glycerol and three fatty acids, triglycerides function as a storage depot for excess lipids [39]. In cattle, triglycerides are measured to determine metabolic status, whereas a buildup of triglycerides in the liver decreases liver function and leads to fatty liver disease [39]. Interestingly, we did observe an effect of day on the circulating concentration of triglycerides, with more variation in quantified concentrations observed under a high estrogenic (d 0) versus progestogenic background (d 7 and d 17). The relationship among estradiol, progesterone, and triglycerides has been routinely investigated. Estradiol implants in mature cows resulted in an elevated concentration of triglycerides on day 14 compared to the controls [40]. This relationship is consistent with previous reports at parturition, particularly in dairy cattle that experience a negative energy balance and mobilize NEFA, BHBA, triglycerides, and very low-density lipoproteins during early lactation [41]. Under the high progestogenic conditions before parturition, triglycerides remain relatively low. However, once progesterone drops dramatically in the periparturient period and estrogen undergoes a steep rise in systemic concentrations, triglycerides have been shown to increase as much as 3-fold by the time of calving compared to before parturition [42,43].

Cholesterol is another substrate closely related to glucose and triglycerides in cattle. Cholesterol is the critical precursor for the production of sex steroids, is vital as a cell membrane constituent, and is a modifier of neuronal signaling molecules [44]. Further, cholesterol is the foundation for the synthesis of bile salts to be excreted into the small intestine to solubilize and convert lipids and fats [45]. Relatedly, cholesterol is packaged in lipoproteins and circulated throughout the body, and binding of low-density lipoprotein to its receptor (LDLR) is the primary way that cholesterol is delivered to steroidogenic luteal cells to serve as substrate for the production of P4 [46]. Herein, total systemic cholesterol was higher in ISe- versus MIX-treated heifers. This is consistent with previous findings of early luteal and gestational concentrations of P4 being higher in MIX versus ISe-treated animals [4,6,47], suggesting the form of Se is increasing the abundance of the LDLR, with increased uptake accounting for lower circulating concentrations of cholesterol.

### 4.3. Global Transcriptomics in ICAR Endometrium

With no panoptic perspective of mRNA transcripts in the ICAR in response to form of Se, and to follow up on previous results from targeted mRNA analyses, we utilized next generation sequencing analysis to perform untargeted transcriptomics and investigate the effects of form of Se on canonical pathways that may be regulating endometrial function to support the elongating conceptus. Results here demonstrate significant effects of the form of Se on carbohydrate metabolism in the ICAR tissue of heifers.

Results from IPA revealed a MIX-induced decrease in transcripts regulating carbohydrate metabolism, and an upregulation of those involved in oxidative phosphorylation. Taken together, it is possible that ICAR may be responding to changes in carbohydrate availability to maintain adequate production of energy. In this case, the greater abundance of mRNA encoding proteins across electron transport and ATP synthase may be accounting for a potential decrease in glucose and other carbohydrate inputs to drive oxidative phosphorylation and the production of ATP.

Interestingly, we observed more abundant expressions of mRNAs encoding the key proteins APOE, MSTN, and SLC1A4 in MIX compared to ISe ICAR samples, and these proteins affect the availability and usability of carbohydrates, fats, and lipid intermediaries [38,48,49]. APOE is involved in the catabolism of triglyceride-rich lipoprotein constituents and is primarily responsible for packaging and transporting cholesterol from the peripheral tissues to be metabolized in the liver and for regulating the availability of glucose to the cell [50]. MSTN has been shown to regulate glucose metabolism in muscle by promoting glucose uptake, supporting glycolysis, and decreasing the storage of glucose as glycogen [38], with increased MSTN also associated with an increase in glucose in the histotroph [14].

The observed greater abundance of mRNA encoding *Slc1a4* in MIX compared to ISe is curious, as this protein (SLC1A4) is a sodium-dependent neutral amino acid transporter that transports alanine, serine, cysteine, proline, and threonine, among others. Interestingly, it also transports glutamine and has been speculated to be involved in the transport of selenoamino acids [51]. Glutamine is the most abundant free amino acid in the muscle tissue of cattle [49], and this is attributed to a potential increase in storage of nitrogen and energy for utilization, particularly in the instance of high metabolic demand [52]. Furthermore, glutamine also acts as a precursor for glutathione, which is necessary for the reduction in harmful ROS [52]. Both of the aforementioned provide a plausible link among the greater abundance of mRNA encoding *Slc1a4* in the ICAR, changes in carbohydrate metabolism, and longer conceptuses of MIX versus ISe heifers.

The observed MIX-induced decrease in abundance of mRNA encoding the proteins ALDOB, PYGL, and MANBA is also relevant. The reversible conversion of fructose-1,6-bisphosphate to glyceraldehyde 3-phosphate and dihydroxyacetone phosphate, affecting glycolysis, is catalyzed by ALDOB [53]. PYGL catalyzes the cleavage of alpha-1,4-glycosidic bonds to release glucose-1-phosphate and is vital for glucose homeostasis [54]. MANBA is a glycosidase involved in catalyzing the removal of mannose monosaccharides from glycans, which collectively may slow the mobilization of glucose from glycogen stores [55].

It appears that the changes to carbohydrate metabolism may allow more substrate, particularly glucose, to be available to histotroph and thus the developing conceptus. Additionally, in response to changes in carbohydrate metabolism, the ICAR may be up-regulating oxidative phosphorylation as indicated by the RNA-sequencing results to account for the lesser available inputs to maintain needed levels of ATP. Here, MIX-Se supplementation may allow the endometrium to overcome an increased production of ROS due to upregulation of the electron transport chain. Overall, there are changes in carbohydrate metabolism that positively influence the growth of conceptus from MIX-supplemented heifers when compared to ISe-supplementation alone.

## 5. Conclusions

In investigating the effects of the form of supplemental Se provided to achieve a Se-adequate status on selenoprotein transcripts in the ICAR, key blood metabolites, and the transcriptomic profile of ICAR, we identified marked relationships between previous reports of endometrial changes and longer conceptuses during MRP. Targeted qPCR analysis revealed an effect of treatment on the abundance of one selenoprotein transcript (*Dio2*) during MRP, a finding consistent with the form of Se-induced changes in selenoprotein mRNAs in the caruncular endometrium, but in stark contrast to the multiple mRNAs affected by the form of Se in the CL. Quantifying serum parameters revealed treatment effects (*p* < 0.05) on systemic concentrations of glucose and cholesterol, but not triglycerides. Effects on systemic glucose can be tied to treatment-induced differences in energy availability for conceptus development, while effects on cholesterol appear related to increased uptake for steroidogenesis through the LDLR. Finally, RNASeq analysis confirmed significant Se-induced changes in carbohydrate metabolism and oxidative phosphorylation, again appearing to alter glucose availability in the ICAR and affect the production of ATP and the cell’s ability to overcome oxidative stress. Overall, the form of Se affected serum parameters during the establishment of pregnancy and ICAR transcriptomics during MRP, which appear to support the early development of the conceptus.

## Figures and Tables

**Table 1:** The relative abundance of mRNA transcripts encoding selenoproteins and selenoprotein P receptors in the intercaruncular (ICAR) tissue of heifers supplemented with ISe (n = 6) or MIX (n = 6) ^1^.

Gene ^2^	Gene Name	qPCR ^3^			
		ISe	MIX	SEM	*p*-Value ^4^
*Iodothyronine deiodinases*					
*Dio1*	Iodothyronine deiodinase 1	Unable to be detected			
*Dio2*	Iodothyronine deiodinase 2	1.2178	0.4352	0.2761	0.02
*Dio3*	Iodothyronine deiodinase 3	1.1500	0.8239	0.2169	0.31
*Glutathione peroxidases*					
*Gpx1*	Glutathione peroxidase 1	1.0492	1.0758	0.1357	0.89
*Gpx2*	Glutathione peroxidase 2	1.3249	1.2657	0.3859	0.92
*Gpx3*	Glutathione peroxidase 3	1.0876	1.3000	0.2383	0.54
*Gpx4*	Glutathione peroxidase 4	1.0501	1.2017	0.1055	0.33
*Gpx6*	Glutathione peroxidase 6	1.3284	0.9822	0.3636	0.83
*Thioredoxin reductases*					
*Txnrd1*	Thioredoxin reductase 1	1.0296	1.0015	0.0917	0.83
*Txnrd2*	Thioredoxin reductase 2	1.0658	1.0399	0.1117	0.88
*Txnrd3*	Thioredoxin reductase 3	1.0411	1.0397	0.1250	0.99
*Other selenoproteins*					
*Selenof*	Selenoprotein F	1.0447	1.1489	0.1122	0.53
*Selenoh*	Selenoprotein H	1.0199	0.8302	0.1028	0.22
*Selenoi*	Selenoprotein I	1.0101	1.0082	0.0832	0.99
*Selenok*	Selenoprotein K	1.0202	0.9775	0.0933	0.73
*Selenom*	Selenoprotein M	4.7204	4.4863	2.1965	0.94
*Selenon*	Selenoprotein N	1.0283	0.8790	0.0849	0.24
*Selenoo*	Selenoprotein O	1.0437	0.8585	0.1088	0.26
*Selenop*	Selenoprotein P	1.0361	0.9158	0.1190	0.49
*Selenor*	Selenoprotein R	1.0838	0.8093	0.1803	0.26
*Selenos*	Selenoprotein S	1.0227	1.1723	0.1205	0.40
*Selenot*	Selenoprotein T	1.0519	0.9570	0.1132	0.57
*Selenov*	Selenoprotein V	1.1392	0.7628	0.2257	0.27
*Selenow*	Selenoprotein W	1.1121	0.9271	0.1809	0.64
*Sephs2*	Selenophosphate synthetase 2	1.0458	1.1432	0.1455	0.65
*Selenoprotein P receptors*					
*Lrp2*	LDL receptor related protein 2	1.0913	0.6536	0.1634	0.09
*Lrp8*	LDL receptor related protein 8	1.0196	0.9290	0.1172	0.60
*Tfrc*	Transferrin receptor	1.0294	0.9993	0.1023	0.84

^1^ Treatment (form of Se) was ad libitum at 35 ppm as either inorganic Se (ISe; sodium selenite), or a 1:1 combination (MIX) of ISe and OSe (1:1 sodium selenite and SEL-PLEX). ^2^ Data for 6 transcripts (*Dio2, Gpx6, Selenok, Selenom, Selenor,* and *Selenow)* were natural log transformed due to not being normally distributed. ^3^ Data are expressed as a ratio of MIX relative to ISe. ^4^ *p*-values are associated with Student’s *t*-test using the PROC TTEST procedure of the SAS statistical software package (version 9.4, SAS Institute Inc.). Samples (ICAR) from n = 6 heifers per treatment were used herein.

**Table 2:** Top 10 most highly up- and downregulated DEGs in the ICAR of MIX (n = 6) versus ISe (n = 6)-treated heifers ^1^.

Gene ID	Gene Description	Fold Change	*p*-Value ^2^
*Up regulated in MIX*			
*Muc4*	Mucin 4, cell surface-associated	3.78	<0.0001
*Kcnn4*	Potassium calcium-activated channel subfamily N member 4	3.58	<0.01
*Sp7*	Sp7 transcription factor	3.52	<0.01
*Lpar5*	Lysophosphatidic acid receptor 5	2.91	0.02
*Orm1*	Orosomucoid 1	2.79	0.03
*Nxph4*	Neurexophilin 4	2.74	0.02
*Tmem132d*	Transmembrane protein 132D	2.67	0.02
*Dusp2*	Dual specificity phosphatase 2	2.48	<0.01
*Tmem217*	Transmembrane protein 217	2.42	0.03
*Znf597*	Zinc finger protein 597	2.02	0.03
*Downregulated in MIX*			
*Sycp3*	Synaptonemal complex protein 3	−5.85	<0.001
*Nalcn*	Sodium leak channel, non-selective	−5.51	0.0001
*Cdhr1*	Cadherin-related family member 1	−4.29	0.02
*Ces5a*	Carboxylesterase 5A	−4.27	0.01
*Nkain2*	Sodium/potassium transporting ATPase interacting 2	−3.82	<0.01
*Dlk1*	Delta-like non-canonical Notch ligand 1	−3.76	0.03
*Aard*	Alanine and arginine-rich domain-containing protein	−3.69	0.04
*Majin*	Membrane-anchored junction protein	−3.68	0.01
*Ca12*	Carbonic anhydrase 12	−3.68	<0.01
*Tas2r4*	Taste 2 receptor membrane 4	−3.38	<0.01

^1^ Selenium was supplemented ad libitum at 35 ppm to heifers as either inorganic (ISe; sodium selenite) or a 1:1 combination (MIX) of ISe and OSe (SEL-PLEX). ^2^ For statistical analysis, *p*-values were determined using the Wald test in DESeq2.

**Table 3:** Top 5 canonical pathways identified by IPA of genes differentially expressed from ICAR of heifers supplemented with either ISe (n = 6) or MIX (n = 6) in their vitamin/mineral mixes ^1^.

Canonical Pathway ^2^	Gene Symbols	Ratio ^3^	Z-Score	*p*-Value
Mitochondrial Dysfunction	**Up:** *Atp5me*, *Atp5mg*, *Cacna1s*, *Clic2*, *Cox6a1*, *Cox7b*, *Cox8a*, *Gsr*, *Map3k5*, *Ndufb6*, *Ndufb11*, *Ndufv3*, *Ppif*, *Prdx6*, *Tomm22*, *Tp53*, *Uqrc10* **Down:** *C9orf72*, *Cacna1d*, *Hap1*, *Mgst3*, *Prkar2a*, *Snca*	0.07 (23/345)	−1.279	<0.0001
Cardiac-adrenergic signaling	**UP:** *Cacna1s*, *Gna15* **Down:** *Adcy2*, *Adcy8*, *Akap6*, *Cacna1d*, *Gnal*, *Gnaq*, *Palm2akap2*, *Pde10a*, *Pde6a*, *Ppp1r12a*, *Ppp1r1a*, *Prkar2a*	0.08 (14/180)	−1.667	<0.0001
Endocannabinoid neuronal synapse pathway	**Up:** *Cacna1s*, *Gna15*, *Mgll*, *Plcb2* **Down:** *Adcy2*, *Adcy8*, *Cacna1d*, *Gnal*, *Gnaq*, *Gria1*, *Mapk13*, *Prkar2a*	0.08 (12/149)	0.302	<0.0001
G beta gamma signaling	**Up:** *Cacna1s*, *Gna15*, *Mgll*, *Plcb2* **Down:** *Adcy2*, *Adcy8*, *Cacna1d*, *Gnal*, *Gnaq*, *Gria1*, *Mapk13*, *Prkar2a*	0.09 (11/129)	−0.905	<0.0001
Synaptogenesis signaling pathway	**Up:** *Apoe*, *Efnb1*, *Epha3*, *Ephb6*, *Rab3a*, *Rab5c*, *Syt17* **Down:** *Adcy2*, *Adcy8*, *Chn1*, *Epha5*, *Epha7*, *Gria1*, *Nrxn1*, *Prkar2a*, *Snap25*, *Snca*, *Thbs2*	0.08 (11/132)	−0.943	<0.0001

^1^ Selenium was supplemented to heifers ad libitum at 35 ppm as either inorganic (ISe; sodium selenite) or a 1:1 combination (MIX) of ISe and OSe (SEL-PLEX). ^2^ Results from IPA were obtained based on log2 fold changes calculated using DESeq2. ^3^ The ratio is calculated as the number of differentially expressed genes (*p* < 0.05) in a given pathway divided by the total number of genes that make up that pathway.

**Table 4:** Top 5 molecular and cellular functions in ICAR of heifers supplemented with either ISe (n = 6) or MIX (n = 6) in their vitamin/mineral mixes, as identified by IPA ^1^.

Molecular and Cellular Functions ^2^	Z-Score ^3^	*p*-Value ^4^
**Cellular assembly and organization (133 molecules)**		
Formation of cellular protrusions	0.726	<0.0001
Neuritogenesis	0.907	<0.0001
**Cellular function and maintenance (207 molecules)**		
Cellular homeostasis	1.424	<0.0001
Formation of cellular protrusions	0.726	<0.0001
Organization of cells	0.659	<0.0001
Neuritogenesis	0.907	<0.0001
Exocytosis	0.636	<0.0001
T cell homeostasis	2.259	<0.0001
**Cell morphology (99 molecules)**		
Formation of cellular protrusions	0.726	<0.0001
Morphogenesis of neurons	0.907	<0.0001
Neuritogenesis	0.907	<0.0001
Cell spreading	0.937	0.0007
**Carbohydrate metabolism (68 molecules)**		
Uptake of monosaccharide	−1.558	0.0002
Uptake of D-glucose	−1.175	0.0003
**Molecular transport (149 molecules)**		
Transport of ions	−0.950	<0.0001
Transport of molecules	−1.640	<0.0001
Transport of metal	−0.855	0.0002
Transport of inorganic cations	0.555	0.0002
Transport of metal ions	0.555	0.0002
Uptake of D-glucose	−1.175	0.0003
Quantity of polyunsaturated fatty acids	0.849	0.0003
Concentration of eicosanoid	1.254	0.0003
Exocytosis	0.636	0.0004

^1^ Selenium was supplemented ad libitum at 35 ppm as either inorganic (ISe; sodium selenite) or a 1:1 combination (MIX) of ISe and OSe (SEL-PLEX). ^2^ Results from IPA were obtained based on log2 fold changes calculated using DESeq2. ^3^ Z-score is calculated using IPA and is used to infer activation state. Only functions reporting a z-score with an absolute value ≥ 0.5 are reported herein. ^4^ *p*-values are determined using IPA.

**Table 5:** Real-time PCR of selected mRNAs identified by RNA sequencing from ICAR samples during MRP of heifers supplemented with either ISe (n = 6) or MIX (n = 6) in their respective vitamin/mineral mixes ^1^.

	RNA-Seq ^3^			qPCR ^3^			
Gene ^2^	ISe	MIX	*p*-Value	ISe	MIX	SEM	*p*-Value ^4^
*Carbohydrate metabolism*							
*Adcy2*	1.00	0.77	<0.01	1.07	0.39	0.15	<0.01
*Aldob*	1.00	0.79	<0.05	1.45	0.52	0.84	0.92
*Apoe*	1.00	1.39	<0.01	1.13	2.53	0.57	0.10
*Bmp4*	1.00	1.18	0.02	1.04	1.00	0.14	0.80
*Edn1*	1.00	1.18	<0.05	1.01	1.34	0.11	0.07
*Gaa*	1.00	1.26	<0.01	1.04	0.73	0.08	0.04
*Gnaq*	1.00	0.91	<0.05	1.02	0.75	0.08	0.04
*Manba*	1.00	0.70	<0.01	1.19	0.43	0.20	<0.05
Mstn *	1.00	1.35	<0.001	1.03	1.71	0.14	0.01
*Neu3*	1.00	1.13	0.03	1.02	0.67	0.07	0.01
*Pygl*	1.00	0.86	0.034	1.01	0.71	0.06	0.01
*Sgsh*	1.00	1.23	<0.01	1.02	0.81	0.48	0.09
*Slc1a4*	1.00	1.42	<0.0001	1.03	1.38	0.15	0.13
*Spp1*	1.00	0.73	<0.01	1.25	0.48	0.30	<0.05
*Canonical Wnt signaling pathway*							
*Cpt1a*	1.00	0.85	<0.05	1.31	0.55	0.28	0.09
*Ctnnb1*	1.00	0.95	0.08	1.01	0.94	0.13	0.73
Dkk1 *	1.00	0.83	0.21	1.10	0.64	0.15	0.05
*Fzd6*	1.00	0.88	0.03	1.03	0.86	0.09	0.23
*Lrp5*	1.00	1.02	0.79	1.01	0.72	0.07	0.01
*Lrp6*	1.00	0.75	0.03	1.02	0.55	0.08	<0.01

^1^ Selenium was supplemented ad libitum at 35 ppm as either inorganic (ISe; sodium selenite) or a 1:1 combination (MIX) of ISe and OSe (SEL-PLEX). ^2^ Data for four transcripts (*Aldob, Apoe, Bmp4,* and *Spp1)* were natural log transformed due to not being normally distributed. ^3^ Data are expressed as a ratio of MIX relative to ISe, and *p*-values are associated with statistical significance between treatment groups (form of Se) for each respective test. * qPCR results are reported in Crites et al. [13], and *p*-values were obtained from one-way ANOVA using the PROC GLM procedure of SAS statistical software package (version 9.4; SAS Institute, Inc.) with n = 6 heifers per treatment.

## Data Availability

The raw data (FASTQ files) are available in the National Center for Biotechnology Information (NCBI) Sequence Read Archive (SRA, accession number PRJNA1096973).

## Appendix A

**Table A1:** Primer sets and product identities of reference transcripts for qPCR analysis.

Gene	Gene Name	Accession Number ^1^	Oligonucleotide Primer Design (5′ to 3′) Direction	Amplicon Length (bp)	Product Identity (%) ^2^
*Reference transcripts*					
*Actb*	Actin beta	NM_173979.3	F: GAGCGGGAAATCGTCCGTGAC R: GTGTTGGCGTAGAGGTCCTTGC	278	99
*Gapdh*	Glyceraldehyde-3-phosphate dehydrogenase	NM_001034034.2	F: ACATCAAGTGGGGTGATGCT R: GGCATTGCTGACAATCTTGA	289	99
*Hprt1*	Hypoxanthine phosphoribosyltransferase 1	NM_001034035.2	F: GCCAGCCGGCTACGTTAT R: ATCCAACAGGTCGGCAAAGA	256	100

^1^ These contents are associated with each gene symbol and are the accession numbers of the sequences retrieved from the NCBI RefSeq database for designing primers and probes. ^2^ All qPCR products were validated by sequencing. The identity values (%) presented are the base pair ratios between the total amplicon length and the number of identical base pairs.

**Table A2:** Primer sets and product identities for qPCR analysis of selenoprotein and selenoprotein P transcripts.

Gene	Gene Name	Accession Number ^1^	Oligonucleotide Primer Design (5′ to 3′) Direction	Amplicon Length (bp)	Product Identity (%) ^2^
*Enzymatic transcripts*					
*Dio1*	Iodothyronine deiodinase 1	NM_001122593.2	F: TCCTGTAGTCCGCCTGTCA R: TCCGGTGATTCTTGATGTCCA	242	99
*Dio2*	Iodothyronine deiodinase 2	NM_001010992.4	F: GATGGGCATCCTCAGCGTAG R: TTCTCCTGGGCACCATTTCC	315	100
*Dio3*	Iodothyronine deiodinase 3	NM_001010993.3	F: AAGTGGAGCTCAACAGCGAT R: AGTCGAGGATGTGCTGGTTC	213	100
*Glutathione peroxidases*					
*Gpx1*	Glutathione peroxidase 1	NM_174076.3	F: GCAACCAGTTTGGGCATCAG R: TAGGGTCGGTCATGAGAGCA	210	100
*Gpx2*	Glutathione peroxidase 2	NM_001163139.2	F: AACAGCCTCAAGTACGTCCG R: TCGGTCATGAGGGAAAACGG	158	100
*Gpx3*	Glutathione peroxidase 3	NM_174077.5	F: GCACCATCTATGAGTACGGGG R: CCCCATTCACATCGCCTTTC	315	100
*Gpx4*	Glutathione peroxidase 4	NM_174770.3	F: GATCAAAGAGTTCGCCGCTG R: CCATACCGCTTCACCACACA	198	100
*Gpx6*	Glutathione peroxidase 6	NM_001163142.1	F: CACTGTTCCTGGTCGGCTTA R: CCCAGCACAACTACACCGAA	259	100
*Thioredoxin reductases*					
*Txnrd1*	Thioredoxin reductase 1	NM_174625.5	F: AAGGCCGCGTTATTTGGGTA R: CCTGGTGTCCCTGCTTCAAT	306	100
*Txnrd2*	Thioredoxin reductase 2	NM_174626.2	F: CAAATGGCTTCGCTGGTCAC R: TTCGTATGCACACCAGCCTT	230	100
*Txnrd3*	Thioredoxin reductase 3	XM_015468824.1	F: CGGCGTATGACTACGACCTC R: GACTGTACTCCCAGCCGAAC	249	100
*Other selenoproteins*					
*Selenof*	Selenoprotein F	NM_001034759.2	F: GCAGCTCCTGTGATTTGCTT R: TTTAGCACAGGGTCTGAACCG	241	100
*Selenoh*	Selenoprotein H	NM_001321327.1	F: CACGAGCTGACGAGTCTACG R: CTTCTTCAGCTCCTCCAGCA	235	100
*Selenoi*	Selenoprotein I	NM_001075257.2	F: TCTGGCTTTCTGCTGGTTGT R: TGGTCAAAAAGCTCCCCCAG	212	100
*Selenok*	Selenoprotein K	NM_001037489.3	F: CCGTTTTGTCGATTCACGGC R: CAGATGAGCTTCCGTAGCCT	278	100
*Selenom*	Selenoprotein M	NM_001163171.2	F: CCCACTCTACCACAACCTGG R: ACCTAAAGGTCTGCGTGGTC	249	100
*Selenon*	Selenoprotein N	NM_001114976.2	F: GTGGCCATGTACCCCTTCAA R: GGGATGGGTTCTCCTGGTTG	265	100
*Selenoo*	Selenoprotein O	NM_001163193.2	F: TGGACAGGTATGACCCCGAT R: ATCTTCTGCAGGTAGTGCCG	202	100
*Selenop*	Selenoprotein P	NM_174459.3	F: TCAGGTCTTCATCACCACCA R: GTGGCAACAGCAGCTACTCA	201	100
*Selenor*	Selenoprotein R, Methionine sulfoxide reductase B1 (MSRB1)	NM_001034810.2	F: GAACCACTTTGAGCCGGGTA R: GGCCATCGTTCAGGAACTCA	221	100
*Selenos*	Selenoprotein S	NM_001046114.3	F: CCCACCCTCGAGACCGA R: GCCCAGGACTGTCTTCTTCC	394	100
*Selenot*	Selenoprotein T	NM_001103103.2	F: TGGTCACCTTCCATCCATGC R: AAGAGGTACAACGAGCCTGC	240	100
*Selenov*	Selenoprotein V	NM_001163244.2	F: ACTCCATTGGCCACCGATTT R: AGGCCACAGTAAACCACTCG	224	100
*Selenow*	Selenoprotein W	NM_001163225.1	F: AGTGTTCGTAGCGGGAAAGC R: CGCGAGAACATCAGGGAAGG	233	98
*Selenophosphate synthetase*					
*Sephs2*	Selenophosphate synthetase 2	NM_001114732.2	F: GATCCCTACATGATGGGGCG R: GTTTACCACCGTTTGCCCAC	219	100
*Selenoprotein P receptors*					
*Lrp2*	LDL receptor related protein 2	XM_024983502.1	F: GTGGTTTGGGTTACCGTTGC R: GGCACCCTGTTAGCTGTGAT	304	99
*Lrp8*	LDL receptor related protein 8	NM_001097565.1	F: AGCCACCCTTTTGGGATAGC R: AAGGCACAGGTACTCACAGC	231	100
*Tfrc*	Transferrin receptor	NM_001206577.1	F: CCAGGTTTAGTCTGGCTCGG R: GGTCTGCCCAGAATATGCGA	339	99

^1^ These contents are associated with each gene symbol and are the accession numbers of the sequences retrieved from the NCBI RefSeq database for designing primers and probes. ^2^ All qPCR products were validated by sequencing. The identity values (%) presented are the base pair ratios between the total amplicon length and the number of identical base pairs.

**Table A3:** Primer sets and product identities for qPCR analysis of transcripts used to corroborate RNA-sequencing results.

Gene	Gene Name	Accession Number ^1^	Oligonucleotide Primer Design (5′ to 3′) Direction	Amplicon Length (bp)	Product Identity (%) ^2^
*Carbohydrate Metabolism*					
*Adcy2*	PREDICTED: Adenylate cyclase 2	XM_024981538.1	F: ATCAGCACCACGGATGTACC R: GAAGATCAGGCAAGCGCAAG	267	99
*Aldob*	Aldolase, fructose-bisphosphate B	NM_001034485.2	F: CAGTTCCGCGAACTCCTCTT R: AGCGTTCAGAAAGGCCATCA	232	100
*Apoe*	Apolipoprotein E	NM_173991.2	F: ACGCTGACGACCTGAAGAAG R: CCTCTAGCTGCTGGCGTATC	261	100
*Bmp4*	Bone morphogenetic protein 4	NM_001045877.1	F: ACTTCGAGGCCACACTTCTG R: AGAGTTTTCGCTGGTCCCTG	245	100
*Edn1*	Endothelin 1	NM_181010.2	F: CTTCTAGGTCCAAGCGCTCC R: CTGATGGCCTCCAACCTTCTT	259	99
*Gaa*	Alpha glucosidase	NM_173913.2	F: CATGACTTGGAGGTGGTCCC R: GTGGTGGTCAGGTTCTCCAG	308	100
*Gnaq*	Guanine nucleotide binding protein (G protein), q polypeptide	NM_001110002.1	F: CGAGCACAATAAGGCTCATGC R: TTGTTGCGTAGGCAGGTAGG	223	100
*Manba*	Mannosidase beta	NM_174387.2	F: CAGTCGCAGCGAGATAGTGA R: TCTGAGTGAAGGTGTGACGC	22	99
*Mstn **	Myostatin	NM_001001525.3	F: TGCCCACGGAGTCTGATCTT R: TGCCTGGGTTCATGTCAAGT	237	100
*Neu3*	Neuraminidase 3	NM_174122.3	F: ATGGAGGAGCCGGGGTT R: CAGGGTTCCTGCCTGACATA	400	100
*Pygl*	Glycogen phosphorylase L	NM_001075203.2	F: GAAGTGCCCCAAGAGGGTTT R: ATGGAATCCAGGAAGCAGGC	213	100
*Sgsh*	N-sulfoglucosamine sulfohydrolase	NM_001102189.2	F: ACAACAGCGCCATCTCTACC R: AGGAATTTCCGGACCAGCAG	367	99
*Slc1a4*	Solute carrier family 1 member 4	NM_001081577.1	F: AGTGACCTACAACACGAGCG R: ACATGATGCCCACAGGTACG	233	100
*Spp1*	Secreted phosphoprotein 1	NM_174187.2	F: GCCTGACCCATCTCAGAAGC R: TCTGAACGTTAGATCGGCGG	386	99
*Canonical Wnt Signaling*					
*Cpt1a*	Carnitine palmitoyltransferase 1A	NM_001304989.2	F: GGGTCTACGATTCCGCTCTG R: GGCATCCAGAGACTGCTTGT	339	100
*Ctnnb1*	Catenin beta 1	NM_001076141.1	F: CAGCAGTTTGTGGAGGGAGT R: GAACTGGTCAGCTCAACCGA	371	100
*Dkk1 **	Dickkopf WNT signaling pathway inhibitor 1	NM_001205544.1	F: GGCAGCAAGTACCAGACCAT R: AGAAGGCATGCATATCCCGTT	207	100
*Fzd6*	PREDICTED: Frizzled class receptor 6, transcript variant X1	XM_010812158.3	F: TGCACAGAATGGGCTGGATT R: GTACCATGATTTGCCGTCGC	212	100
*Lrp5*	PREDICTED: LDL receptor related protein 5, transcript variant X1	XM_024987583.1	F: CTGGAGGAGTTCTCAGCCCAC R: TTCAGAGAGGCGTCGCAGTC	386	100
*Lrp6*	PREDICTED: LDL receptor related protein 6, transcript variant X1	XM_005207028.4	F: GTGCCCTGGAACATGTGGTA R: TTCACGGTTGAGTCCAAGCA	398	100

^1^ These contents are associated with each gene symbol and are the accession numbers of the sequences retrieved from the NCBI RefSeq database for designing primers and probes. ^2^ All qPCR products were validated by sequencing. The identity values (%) presented are the base pair ratios between the total amplicon length and the number of identical base pairs. * Primer pair published in Crites et al. [13]

## Appendix B

**Table A4:** Gene transcripts associated with carbohydrate metabolism that were affected by form of selenium (MIX compared to ISe).

ID	Gene	Expr Log Ratio
ENSBTAG00000019988	*GNA15*	1.284
ENSBTAG00000002982	*PITPNM3*	1.041
ENSBTAG00000011808	*MSTN*	0.988
ENSBTAG00000004855	*PRDX6*	0.975
ENSBTAG00000018223	*CHI3L1*	0.935
ENSBTAG00000010123	*APOE*	0.784
ENSBTAG00000019079	*PLCB2*	0.656
ENSBTAG00000016021	*GAA*	0.650
ENSBTAG00000004150	*NRG1*	0.612
ENSBTAG00000038062	*FUT4*	0.598
ENSBTAG00000015267	*SGSH*	0.563
ENSBTAG00000018119	*AOAH*	0.527
ENSBTAG00000008096	*EDN1*	0.490
ENSBTAG00000046155	*RGN*	0.433
ENSBTAG00000037527	*OAS1*	0.386
ENSBTAG00000001420	*ABHD12*	0.354
ENSBTAG00000000134	*MPDU1*	0.349
ENSBTAG00000030434	*FUCA1*	0.328
ENSBTAG00000010336	*TALDO1*	0.326
ENSBTAG00000021504	*TIRAP*	0.322
ENSBTAG00000014534	*EEF1A1*	0.303
ENSBTAG00000025931	*NEU3*	0.294
ENSBTAG00000010136	*CMAS*	0.280
ENSBTAG00000004587	*DUSP6*	0.273
ENSBTAG00000011056	*IDS*	0.258
ENSBTAG00000005371	*DPAGT1*	0.257
ENSBTAG00000016845	*GALNT7*	0.234
ENSBTAG00000009789	*GNAQ*	−0.185
ENSBTAG00000011709	*IMPA1*	−0.203
ENSBTAG00000034436	*PDPK1*	−0.204
ENSBTAG00000011494	*PYGL*	−0.291
ENSBTAG00000005997	*ABCB1*	−0.296
ENSBTAG00000011761	*LRP6*	−0.348
ENSBTAG00000021999	*CPT1A*	−0.423
ENSBTAG00000005359	*TGFB2*	−0.426
ENSBTAG00000050602	*HAS3*	−0.540
ENSBTAG00000001879	*PER2*	−0.556
ENSBTAG00000010206	*UAP1*	−0.579
ENSBTAG00000000507	*NR4A1*	−0.591
ENSBTAG00000002699	*KIT*	−0.630
ENSBTAG00000023600	*APOD*	−0.631
ENSBTAG00000019026	*EXTL2*	−0.634
ENSBTAG00000014674	*CHRM2*	−0.745
ENSBTAG00000001649	*ZFPM2*	−0.779
ENSBTAG00000019761	*MANBA*	−0.841
ENSBTAG00000024957	*SNCA*	−0.903
ENSBTAG00000019892	*HAS2*	−0.961
ENSBTAG00000019210	*ADCY2*	−1.030
ENSBTAG00000015358	*ALDOB*	−1.077
ENSBTAG00000005260	*SPP1*	−1.311
